# Diagnostic Yield of FDG-PET/CT, MRI, and CSF Cytology in Non-Biopsiable Neurolymphomatosis as a Heralding Sign of Recurrent Non-Hodgkin's Lymphoma

**DOI:** 10.7759/cureus.319

**Published:** 2015-09-09

**Authors:** Faiq Shaikh, Aubrey C Chan, Omer Awan, Nivedita Jerath, Chandan Reddy, Salman A Khan, Michael M Graham

**Affiliations:** 1 Imaging Informatics, University of Pittsburgh Medical Center; 2 Molecular Imaging Physician, S&L Readings, LLC.; 3 CEO, Crunchtimr Medical Solutions, LLC; 4 Department of Internal Medicine, University of Iowa Hospitals and Clinics; 5 Department of Radiology, Dartmouth Hitchcock Medical Center; 6 Department of Neurology, University of Iowa Hospitals and Clinics; 7 Department of Neurosurgery, University of Iowa Hospitals and Clinics; 8 Department of Internal Medicine, University of Missouri Kansas City; 9 Department of Radiology, University of Iowa Hospitals and Clinics

**Keywords:** mri, pet-ct, fdg, non-hodgkin's lymphoma, csf cytology, neurolymphomatosis, diffuse large b-cell lymphoma (dlbcl)

## Abstract

Neurolymphomatosis (NL) is a rare condition associated with lymphomas in which various structures of the nervous system are infiltrated by malignant lymphocytes. Rarely, it may be the presenting feature of recurrence of lymphoma otherwise deemed to be in remission. It is crucial, as is the case with all types of nodal or visceral involvement of lymphoma, to identify the disease early and initiate treatment with chemotherapy and/or radiation therapy. Positron emission tomography-computed tomography (PET-CT) has been shown to be a sensitive modality for staging, restaging, biopsy guidance, therapy response assessment, and surveillance for recurrence of lymphoma. Magnetic resonance imaging (MRI) is another useful imaging modality, which, along with PET/CT, compliment cerebrospinal spinal fluid (CSF) cytology and electromyography (EMG) in the diagnosis of NL. Performing nerve biopsies to confirm neurolymphomatosis can be challenging and with associated morbidity. The case presented herein illustrates the practical usefulness of these tests in detecting NL as a heralding feature of lymphoma recurrence, especially in the absence of histopathologic correlation.

## Introduction

We present a rare case of a patient with neurolymphomatosis as a heralding sign of recurrent Non-Hodgkin's lymphoma, which became a challenging diagnosis given its clinical obscurity and the technical challenges and morbidity associated with performing a nerve biopsy. Subsequently, the diagnosis was based on non-interventional modalities, such as FDG-PET/CT, MR imaging, and cerebrospinal cytology.

## Case presentation

Informed patient consent was obtained for this patient's treatment.

**Figure 1:**
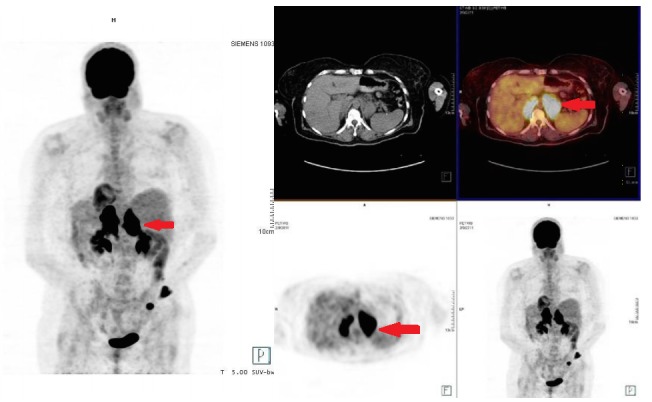
Staging FDG-PET/CT FDG-avid bilateral retroperitoneal lymphadenopathy is visualized (see arrows) indicating sites of original lymphomatous involvement.

A 51-year-old woman presented with a painless cutaneous mass over her anteromedial left ankle that had been present for several months, slowly growing in size. This mass was surgically excised, and pathology results revealed diffuse large B-cell lymphoma (DLBCL), for which she was referred to our hematology-oncology service. The approximate size of the mass at the time of excision was 4.0 x 3.5 cm. Initial staging PET/CT revealed bulky, hypermetabolic, confluent lymphadenopathy in the retroperitoneum (Figure 1). She was therefore diagnosed with Stage IV DLBCL, with an International Prognostic Index of 2 (for Stage IV and elevated lactate dehydrogenase (LDH)). Bone marrow biopsy at the time showed no evidence of lymphoma.

**Figure 2:**
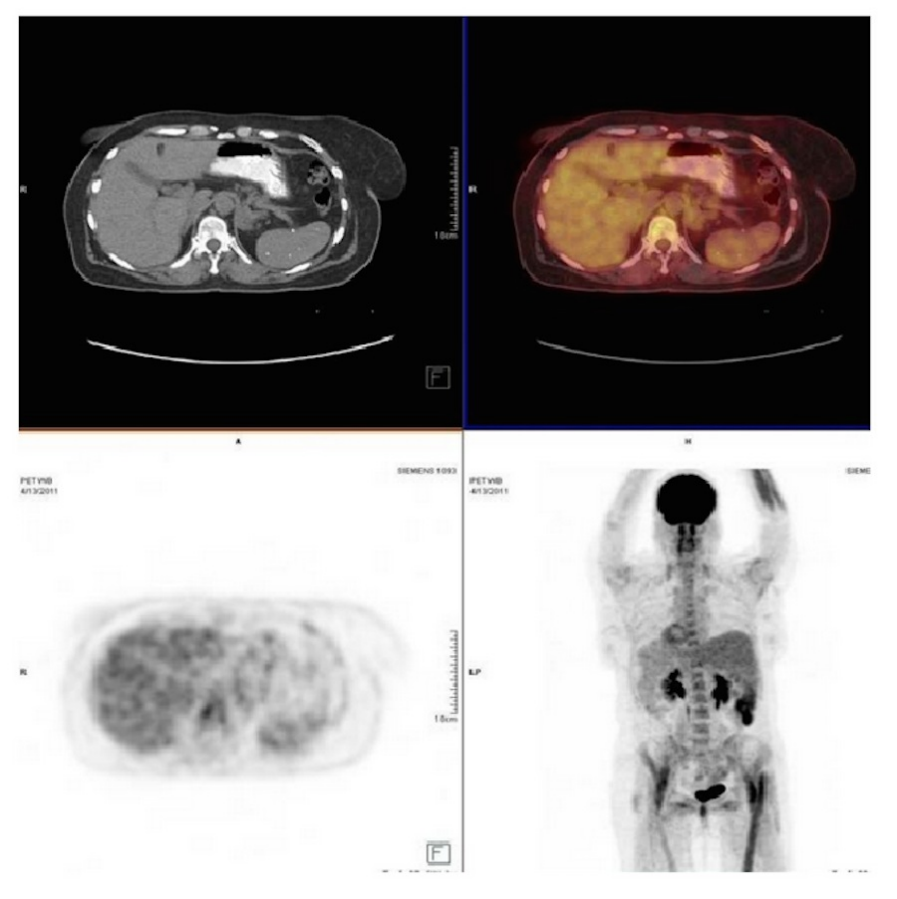
Post-therapy FDG-PET/CT No evidence of FDG-avid nodal lesions suggestive of resolution of previously noted FDG-avid lymphomatous disease.

She received six cycles of rituximab, cyclophosphamide, hydroxydoxorubicin, vincristine, and prednisone (R-CHOP). Repeat PET-CT after her third cycle (after four months) showed resolution of the retroperitoneal lymphadenopathy (Figure 2). She received radiation therapy to her left ankle as well. Surveillance CT scans at 12 and 16 months after her last cycle of chemotherapy showed no evidence of lymphadenopathy. At a routine follow-up appointment 35 months after her last chemotherapy cycle, she presented with a history of a one month-long right leg weakness, multiple falls, pain in her right buttock radiating down the leg, rectal pain, constipation, and weight loss. She was admitted to the hospital for further workup with concern for recurrence of lymphoma.

Magnetic resonance imaging (MRI) at this time showed thickening and enhancement of the right L5 nerve root; however, the PET-CT was negative for hypermetabolic lesions. Two lumbar punctures were performed with negative cytology from both samples of cerebrospinal fluid (CSF). Electromyography and nerve conduction studies (EMG/NCS) were performed, which were consistent with L5 radiculopathy as suggested by a significantly reduced amplitude in the right tibial and peroneal and bilateral sural nerves, as well as absent H-wave responses. Biopsy of the nerve root was considered but deferred due to concern for resultant paresis or paralysis. She was treated with a course of dexamethasone, discharged ambulating with a four-wheeled walker, and was prescribed pain medications.

**Figure 3:**
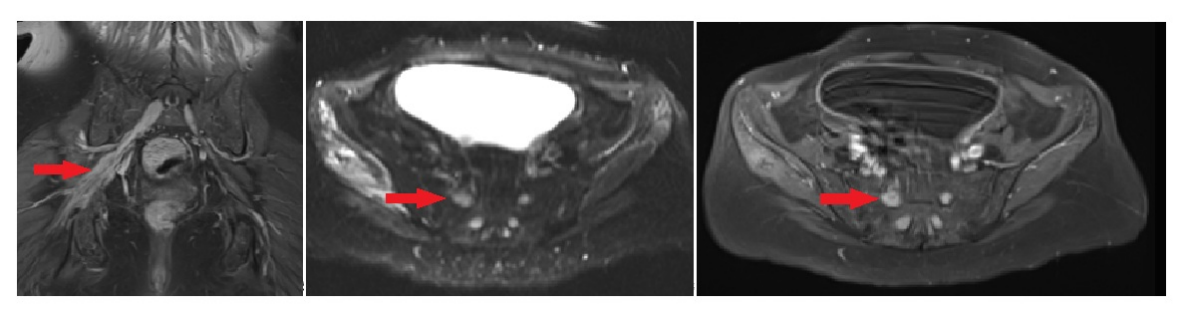
MRI of pelvis T1-weighted coronal T1 images and axial post-contrast images demonstrate S1 and sacral plexus enlargement (see arrow), and T2-weighted axial images demonstrate hyperintense signal within the right gluteus minimus and medius musculature.

Four months later, a follow-up MRI was performed while the patient was symptom-free, which showed stable enhancement of nerve roots (Figure 3).

**Figure 4:**
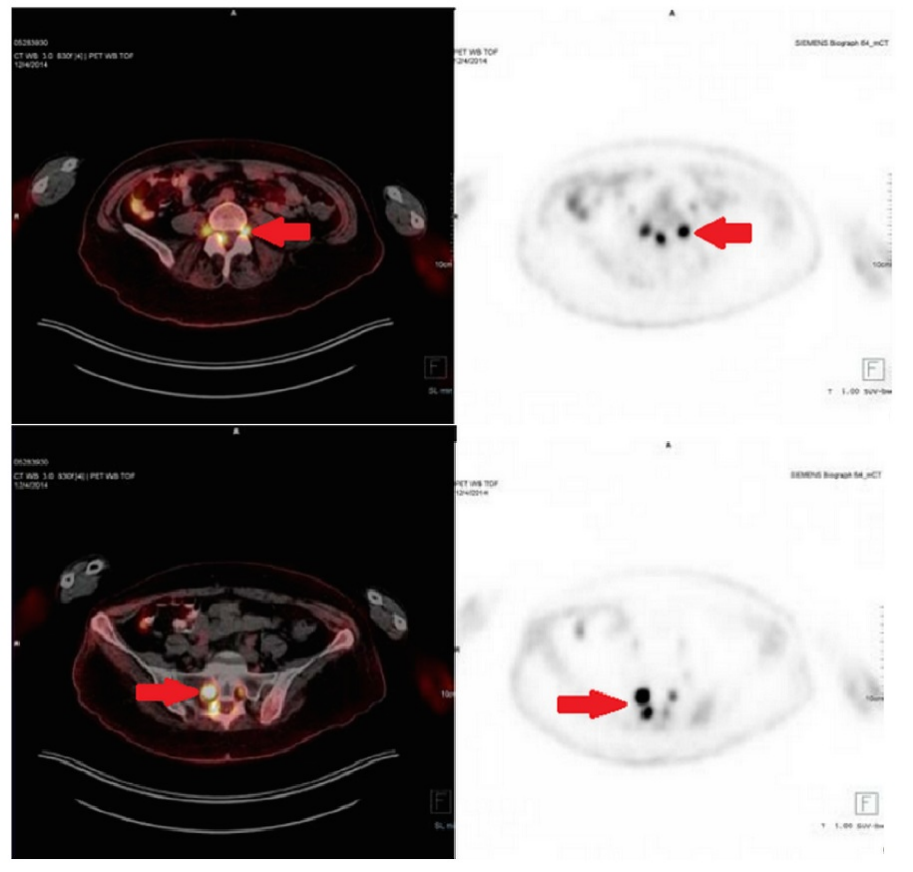
FDG-PET/CT for recurrence assessment FDG-avid involvement of the left T1, bilateral lumbar and sacral nerve roots as well as the right sacral plexus and right sciatic nerve (see arrows).

However, two months later, she began to experience progressive weakness of both legs and numbness of the right leg with severe bilateral pain starting in the buttocks and radiating down the legs. She also experienced severe weakness of the legs with complete inability to bear weight, diminished sensation across the legs, and diminished leg reflexes, all of which were worse on the right than the left, as well as intermittent urinary incontinence. Cranial nerve function and upper extremity strength and sensation were fully intact. Repeat EMG/NCS showed axonal sensorimotor polyneuropathy, worse on the right than the left. PET-CT showed conspicuous FDG uptake in the thickened left nerve root of T1, right lateral spinal canal at L4 level, and CT evidence of thickened bilateral L4, L5 and sacral nerve roots, sacral plexus, and the right sciatic nerve throughout its length extending to the mid-thigh level, suggestive of lymphomatous involvement (Figure 4). Nerve biopsy was again deferred due to concern for resultant paralysis. A lumbar puncture was again performed. This time, cytology showed atypical lymphocytes suspicious for lymphoma. Flow cytometry performed on the CSF showed CD45+, CD19+, CD20+, CD200+, CD5-, CD10-, and CD23- medium to large-sized cells that were positive for FMC-7 with lambda restriction. Bone marrow biopsy was again performed and showed no evidence for lymphoma.

The nerve biopsy was not performed, given the depth of the lesions and the inherent risk of nerve damage leading to neurological deficit. However, the diagnosis of neurolymphomatosis as a feature of the recurrence of DLBCL was made based on the PET/CT, MRI, and CSF cytology.

## Discussion

Neurolymphomatosis is a rare manifestation of hematologic malignancy characterized by infiltration of the cranial nerves, peripheral nerves, nerve roots, or nervous plexuses by malignant lymphocytes [1]. It causes progressive and painful axonal polyneuropathy. It may occur as a first manifestation of non-Hodgkin lymphoma (NHL), sequelae of widespread NHL, or as a sole relapse site of NHL. In 50% of cases, it is associated with leptomeningeal involvement [2]. Approximately 80% of reported NL cases originate from B-cells, most of which involve aggressive lymphoma [1]. Non-Hodgkin lymphoma (NHL) infiltrates the nervous system in approximately 10-25% of cases; however, peripheral neuropathy as a complication of lymphoma is reported to be anywhere from 0.1 - 10% of patients [3-6].

Diagnosis of neurolymphomatosis can be challenging as a nerve biopsy can be technically difficult to target the patchy lesions of the disease and associated disabilities. FDG-PET/CT is a sensitive imaging modality for the diagnosis [1, 7]. It was found to be superior to MRI as Grisariu, et al., who retrospectively analyzed 50 patients with NL, found that MRI and FDG-PET/CT were positive in 77 and 84%, respectively. However, CSF cytology was positive in only 40%, while a nerve biopsy was positive in 88% [1]. MRI, CSF cytology, and bone marrow examination may all be negative and attempting to biopsy the involved nerves is associated with sampling errors, given the patchy albeit widespread neural lymphomatous infiltration [3-5]. If the obvious morphological abnormality involves the nerve or nerve root, it can be easily detected by contrast-enhanced, thin-slice T1-weighted coronal MRI images. However, the sensitivity of MRI is often limited because of the patchy distribution or small lesions of the disease resulting in false-negative assessment of the affected neural structures. Conversely, FDG-PET/CT was shown to be sensitive in detecting NL lesions by Zhou, et al., where the most commonly seen regions were the thoracic and lumbar nerve roots, some with bilateral involvement [8]. Other case reports have described other areas of involvement, such as the spinal cord, sciatic nerve, brachial and lumbosacral plexus, sacral nerve root, and vagus nerve, etc. [9-13]. In our patient, the MRI detected sacral plexus and sciatic nerve involvement, which were then confirmed on PET/CT. 

Liesbeth, et al. reported 59% concordance between MRI and PET findings of neurolymphomatosis lesions. PET alone had a 91% accurate detection of lesions in various structures in the central or peripheral nervous system. They reported complete remission in 59% of patients, a partial response in 7%, and progressive disease in 34%. They relied on PET imaging, as other diagnostic modalities, such as CSF cytology or bone marrow examination, were in most cases not helpful in diagnosing neurolymphomatosis. Histological examination confirmed the diagnosis of neurolymphomatosis in nine out of 10 cases [14]. It must be be noted that nerve biopsies may be inconclusive or present high false-negative rates given the inhomogeneous pattern of neural involvement and are assocated with procedural complications, such as paresis/paraplegia. In our patient, CSF cytology confirmed the clinical and radiologic suspicion of neurolymphomatosis and proved to be essential, especially when an image-guided biopsy was precluded, given the high risk of resultant paralysis.

## Conclusions

Neurolymphomatosis, albeit rare, can sometimes present as the only sign of Non-Hodgkins lymphoma recurrence and early detection becomes exceedingly important. When dealing with each diagnostic modality, it is important to recognize their limitation and use them in a complementary fashion to reach a reliable diagnosis. MRI has a high sensitivity but lower specificity in this role, while PET/CT has a higher specificity and can play an important synergistic role, especially since CSF cytology has a high false-negative rate and histologic correlation may be difficult, given the concern for associated procedural complications. Therefore, the combined use of MRI/PET/CT and CSF analysis can lead to the diagnosis of neurolymphomatosis with otherwise resolved NHL/DLBCL in the absence of histopathologic correlation.
